# Planned or recent first consultation with the general practitioner for knee complaints: Is it indicative of early-stage knee osteoarthritis?

**DOI:** 10.1016/j.ocarto.2024.100493

**Published:** 2024-06-06

**Authors:** Nuria EJ. Jansen, Dieuwke Schiphof, Jos Runhaar, Edwin HG. Oei, Sita MA. Bierma-Zeinstra, Marienke van Middelkoop

**Affiliations:** a Department of General Practice, Erasmus MC University Medical Center Rotterdam, the Netherlands; b Department of Radiology & Nuclear Medicine, Erasmus MC University Medical Center Rotterdam, the Netherlands

**Keywords:** Early-stage, Overweight, Primary care, Diagnostic criteria

## Abstract

**Objective:**

No established definition for early-stage knee osteoarthritis (KOA) is available, nor classification criteria. Identifying the characteristics of individuals presenting with early-stage KOA symptoms can enhance diagnosis to prevent progression. This study aimed to describe clinical and structural features of individuals presenting with knee complaints within two years after their first consultation, while exploring differences in the duration of knee complaints.

**Method:**

Baseline data was used from the LITE randomized controlled trial, assessing the effectiveness of a lifestyle intervention for individuals with knee complaints and overweight in primary care. Baseline assessments included questionnaires, clinical assessment, and MRI of the most symptomatic knee. Differences between groups with varying durations of knee complaints (<12, ≥12-<24, ≥24 months) were evaluated.

**Results:**

Participants (N ​= ​218, 65% female, mean age 59 ​± ​6 years, mean BMI 32 ​± ​5 ​kg/m^2^) had a median knee complaint duration of 14 months, with an average KOOS pain score of 60 ​± ​17.46% reported their symptoms as unacceptable. Structural MRI-defined KOA was observed in 71% of participants. There were no significant differences in clinical or structural MRI features between different durations of knee complaints.

**Conclusion:**

Within 24 months of initial consultation, over two-thirds of participants displayed MRI-defined structural KOA, and nearly half reported unacceptable symptom states. This study found no association between the duration of knee complaints and symptoms severity or structural KOA presence, underscoring the complexity of identifying stages of KOA among individuals with overweight. Future studies should explore additional features beyond current considerations to facilitate early-stage KOA diagnosis, specifically for individuals with overweight.

## 1 Introduction

Treatment in an early stage of knee osteoarthritis (KOA) is essential to reduce the disease burden and prevent long-term disability [1,2]. However, treating KOA early is challenging, as its clinical presentation varies, and many individuals either delay or forego seeking medical advice when symptoms appear, thereby delaying diagnosis in primary care [3,4]. Identifying the characteristics of individuals presenting with potential early KOA related symptoms in primary care can enhance timely diagnosis to prevent progression to established KOA.

Several diagnostic criteria (not to be confused with classification criteria) for early-stage KOA have been proposed [[5], [6], [7]] with some validated albeit to a limited extent [8,9]. However, these criteria often involve complex elements such as radiography or consist of many features, limiting their practicality in primary care. Implementing appropriate criteria in primary care facilitates the adoption of more suitable management strategies for early stages, such as education, exercise, and weight loss [10], to mitigate risk factors and prevent progression. Promising clinical features for the identification of early-stage KOA have been suggested [1,11,12]. These include assessment of physical functioning, pain characteristics such as duration or pattern, along with findings from clinical examination including joint line tenderness, stiffness, and crepitus [1,11,12]. Additionally, it is important to consider risk factors such as older age, history of knee joint trauma, occupation, and menopause, alongside lifestyle-related features like overweight and physical inactivity [1,11,12]. Furthermore, the absence of other diseases, such as rheumatoid arthritis or psoriatic arthritis, may further support the diagnosis [1,11,12]. To select KOA features and determine appropriate cut-offs for diagnostic criteria, a more comprehensive understanding of the initial presentation of individuals with undifferentiated knee symptoms in primary care is necessary.

General practitioners (GPs) or physiotherapists in primary care often serve as the initial point of contact for individuals with knee complaints, making their role crucial in the early diagnosis of KOA [13]. However, early diagnosis relies on individuals with knee complaints seeking health care in an early stage of the disease. This was clearly shown by Bedson et al. who demonstrated that in a population-based cohort study only 28% of individuals with knee pain consulted their general practitioner (GP) over an 18-month period [4]. Notably, among individuals that had not consulted their GP, more than half had knee complaints persisting for more than 5 years [4]. Research has shown that individuals perceive OA as an inevitable part of ageing that cannot be treated or believe that GPs may hold a similar negative attitude towards OA [[14], [15], [16], [17]]. Consequently, the delay or avoidance of consultation following symptom onset raises concerns that the initial presentation in primary care might be capturing a population more reflective of later-stage rather than early-stage KOA. This emphasizes the importance of considering the duration of knee complaints in the diagnostic process. However, it is unclear if shorter durations of knee complaints may indeed be associated with stages of KOA.

The Lifestyle Intervention Trial for Early-stage KOA (LITE) study aims to evaluate the (cost-)effectiveness of a combined lifestyle intervention (GLI) for individuals presenting with knee complaints and overweight in primary care. The LITE trial recruited individuals who either intended to consult their GP for knee complaints for the first time or already did so within the past 24 months before inclusion. It is imperative to consider the heterogeneity of patient profiles in KOA, including individuals with overweight, who may experience distinct disease and/or illness trajectories. Understanding their clinical and structural characteristics, alongside the duration of knee complaints of the study population is vital in developing a more precise definition of early-stage KOA. The duration of knee complaints is a promising feature that could be applied in diagnostic or classification criteria. Therefore, this study's primary objective is to provide a comprehensive description of the clinical and structural features of individuals presenting with knee complaints and overweight within 2 years of their initial primary care consultation. The secondary objective is to explore whether the duration of knee complaints is associated with these clinical and structural features.

## 2 Method

The LITE study is a randomized controlled trial on the (cost-)effectiveness of a lifestyle intervention for individuals presenting with knee complaints and overweight [18]. Participants were recruited through searches in 27 general practice databases and were approached by their GP. Additional recruitment strategies included advertisements in newspapers, posters in community centers, announcements on the Erasmus MC website, and social media platforms. Participants were eligible for inclusion if they were aged 45–70 years, were overweight (body mass index (BMI)≥25 ​kg/m^2^), were diagnosed by the study staff with clinical KOA according to the National Institute for Health and Care Excellence guidelines (aged 45 or over, activity-related joint pain, and either no morning joint-related stiffness or morning stiffness that lasts no longer than 30 ​min), had no other pathological conditions that could explain the joint complaints, and either intended to consult their GP with knee complaints for the first time or already did so within the previous 24 months. Those who had not yet visited their GP were asked about their intentions, whether they had already made an appointment or were planning to schedule one. Previous GP visits for traumatic knee injuries were allowed, provided these injuries were not the cause of their current complaints. In this study, the term ‘overweight’ encompasses individuals with a BMI greater than 25 ​kg/m^2^, thereby including those classified as obese as well.

The trial received ethical approval from the METC of Erasmus MC University Medical Center Rotterdam (MEC 2020-0943).

### 2.1 Data collection

After participants provided written informed consent, all participants completed a baseline questionnaire and were invited for the baseline visit where clinical assessment and an MRI of the index knee (i.e., most symptomatic knee) was acquired.

### 2.2 Questionnaire

Participants filled out a questionnaire to gather demographic data (age, sex, education) and details about their knee complaints, including the duration of current knee complaints (‘How many months have you had your KOA symptoms?‘) and history of knee injuries (year, cause, surgery) in either knee. The reported duration of knee complaints, as specified by participants at baseline, was used for the primary analysis. Given the interval between the GP visit and baseline assessment, the duration of knee complaints at the first GP consultation was calculated for participants who had already visited their GP, excluding those who intended to do so. Current knee pain during rest and activity was scored using an 11-point Numeric Rating Scale. Other questionnaires included the Knee injury and Osteoarthritis Outcome Score (KOOS) [19], Intermittent and Constant OsteoArthritis Pain [20,21], Patient Acceptable Symptom State [22], Tampa Scale of Kinesiophobia (TSK) [23], and the Self-Administered Comorbidity Questionnaire [24]. KOOS scores were transformed to a 0–100 scale, with zero representing extreme knee problems and 100 representing no knee problems. Intermittent and Constant OsteoArthritis Pain scores were transformed to obtain total scores ranging from 0 (no pain) to 100 (extreme pain). Patient Acceptable Symptom State was determined by a single yes/no question: “If your health was to remain for the rest of your life as it has been during the last 48 ​h, would this be acceptable or unacceptable to you?“. Higher TSK scores indicate greater fear of movement, with a TSK score greater than 37 indicating high kinesiophobia. The Self-Administered Comorbidity Questionnaire was adapted to include additional comorbidities relevant to OA and included a question on drug treatment for hypertension or hyperglycemia. The presence of OA in joints was assessed using a multiple-choice question: “In which other joints besides your knee do you have osteoarthritis?” with options including neck, hip, back, hands and/or fingers, ankles, shoulders, and feet and/or toes. The index knee was assessed for fulfilling the clinical ACR criteria for KOA, which include knee pain and at least three of the following features: age >50 years, stiffness <30 ​min, crepitus, bony tenderness, bony enlargement, and no palpable warmth [25].

### 2.3 Clinical assessment

During the physical examination, body weight, height, and waist circumference at the midpoint between the iliac crest and the lowest rib were measured. BMI was calculated using height and weight (kg/m^2^). Both knees were examined for the presence of joint line tenderness [26] and crepitus with active movement [25]. Systolic and diastolic blood pressure were measured with an oscillometric device (Omron HBP-1320, Omron Health Care Inc., Illinois, USA).

### 2.4 Laboratory assessment

Non-fasting venous blood samples were collected to determine serum concentrations of IL-6 (pg/mL), HDL-cholesterol (mmol/L), and triglyceride (mmol/L) levels.

### 2.5 MRI acquisition and interpretation

MRI of the index knee was acquired on a 1.5T MRI scanner (MAGNETOM Sola, Siemens Medical Solutions, Erlangen, Germany) with a dedicated 18-channel knee coil (see supplement **File S1** for MR Imaging protocol) [18]. The presence of articular cartilage loss, bone marrow lesions, osteophytes, lesions of the menisci, joint effusion, and synovitis was assessed using the semi-quantitative scoring system MRI Osteoarthritis Knee Score (MOAKS) [27]. In MOAKS, a cartilage score of ≥1.0 indicates cartilage defects. BMLs and meniscal extrusion were considered present with a MOAKS grade of ≥1 on a 0–3 scale. Grade ≥2 osteophytes were classified as definite osteophytes. Hoffa-synovitis or effusion-synovitis was defined as a grade of ≥2. Structural severity distribution was assessed by selecting the peak grade as the highest grade assigned to each feature across subregions. See supplement **File S2** for an overview of the inter-observer reliability of the MOAKS scoring, which was 0.77 (95% CI 0.75, 0.80) for all features in all compartments.

### 2.6 Definition of metabolic syndrome

A diagnosis of metabolic syndrome was made when at least three of the following five conditions were met: abdominal obesity: elevated waist circumference (≥80 ​cm for females, ≥94 ​cm for males), dyslipidemia: elevated triglycerides (≥150 ​mg/dL), dyslipidemia: reduced HDL-cholesterol (<50 ​mg/dL for females, <40 ​mg/dl for males), hypertension: elevated BP (systolic ≥130 and/or diastolic ≥85 ​mmHg) or antihypertensive drug treatment (SCQ single-item question), hyperglycemia: drug treatment for elevated glucose (SCQ single-item question) [28].

### 2.7 Definition of structural KOA

The MOAKS subregions were combined to define three articular compartments: patellofemoral (PF; patella: medial and lateral and trochlea: medial and lateral), medial tibiofemoral (TF; medial femoral condyle: central and posterior; medial tibia plateau: anterior, central, and posterior), and lateral TF (lateral femoral condyle: central and posterior; lateral tibia plateau: anterior, central, and posterior). KOA in the PF and medial and lateral TF compartment was defined using a previously proposed definition, detailed in Supplementary File S3 [29].

### 2.8 Statistical analysis

Descriptive statistics stratified by duration of complaints (<12 months, ≥12-<24 months, ≥24 months) were applied to describe participant characteristics and present frequencies of MRI features at baseline. Differences in baseline characteristics between groups were assessed using analysis of variance for continuous variables and chi-squared for categorical variables. For non-parametric continuous variables, the Kruskal-Wallis test was applied, and Fisher's exact test was used for categorical variables with small group sizes. A subgroup analysis explored differences between participants who intended to consult their GP for knee complaints (“planned consultation”) and those who had already done so (“recent consultation”). All analyses were conducted using Rstudio (version 2022.07.2, R Foundation for Statistical Computing, Vienna, Austria) with statistical significance set at p ​< ​0.05.

## 3 Results

The characteristics of the study participants (N ​= ​218) stratified by knee complaints duration are shown in Table 1. Participants had a mean (SD) age of 59 (6) years, a BMI of 32 (5) kg/m^2^, and 65% were female. The overall median [IQR] duration of knee complaints reported at baseline was 14 [9,24] months and when categorized by duration: <12 months (n ​= ​66, 30%), 6 [4,8] months; ≥12-<24 months (n ​= ​81, 37%), 12 [13,18] months; ≥24 months (n ​= ​71, 33%), 34 [24,42] months. Among the 76% of participants who visited their GP for a first consultation, the mean (SD) duration of knee complaints at that initial visit was 11 (7) months, with a median [IQR] duration of 14 [9,24] months at baseline. Participants who intended to visit their GP reported a median [IQR] duration of knee complaints of 12 [6,24] months. Participants reported a mean (SD) KOOS pain severity score of 60 (17), a pain severity during activity of 5 (2), and 46% considered their symptom state unacceptable. In addition to KOA and overweight, participants, on average, presented with two other coexisting conditions (see Supplement Table S4 for a list of reported co-morbidities). A total of 37% of participants reported OA in joints other than the knee (see Supplement Table S5). 92% of the participants met the clinical ACR criteria for KOA in their index knee. No statistically significant differences were observed between the duration of knee complaints and clinical features of knee OA. The characteristics of the study participants, stratified by planned or recent first consultation with the GP, are presented in Supplementary Table S6.

**Table 1:** Characteristics of the study population stratified by duration of knee complaints.

Characteristics	Overall (N=218)	<12 months (n=66)	≥12-<24 months (n=81)	≥24 months (n=71)	Comparison^a^
**Demographics**					
Sex, n (%)					p ​= ​0.38
Female	142 (65.1)	47 (71.2)	53 (65.4)	42 (59.2)	
Male	75 (34.4)	19 (28.8)	28 (33.3)	29 (40.8)	
Intersex	1 (0.5)	0 (0.0)	1 (1.2)	0 (0.0)	
Age, years	59.1 (6.2)	59.9 (5.9)	59.3 (6.2)	58.1 (6.4)	F(2,215) ​= ​1.49, p ​= ​0.23
BMI, kg/m^2^	31.9 (4.5)	32.7 (5.0)	31.4 (4.4)	31.6 (4.2)	F(2,215) ​= ​1.78, p ​= ​0.17
Co-morbidities^b^	2.1 (1.8)	2.2 (1.9)	2.2 (1.8)	1.9 (1.6)	F(2,215) ​= ​0.41, p ​= ​0.66
MetS, n (%)	62 (30.8)	25 (41.0)	22 (29.3)	15 (23.1)	X(2, N ​= ​201) ​= ​4.86, p ​= ​0.09
Education, n (%)					p ​= ​0.63
Primary or secondary	51 (23.4)	12 (18.2)	23 (28.4)	16 (22.5)	
Vocational	77 (35.3)	23 (34.8)	27 (33.3)	27 (38.0)	
Higher	85 (39.0)	30 (45.5)	30 (37.0)	25 (35.2)	
Other	5 (2.3)	1 (1.5)	1 (1.2)	3 (4.2)	
**History and current knee complaints**					
Unilateral knee complaints, n (%)	131 (60.1)	43 (65.2)	42 (51.9)	46 (64.8)	X(2, N ​= ​218) ​= ​3.65, p ​= ​0.16
Previous knee surgery^c^, n (%)	31 (14.2)	8 (12.1)	13 (16.0)	10 (14.1)	X(2, N ​= ​218) ​= ​0.46, p ​= ​0.79
**Patient reported outcomes**					
Knee pain at rest (NRS, 0–10)	3.4 (2.4)	3.4 (2.3)	3.3 (2.5)	3.4 (2.4)	F(2,215) ​= ​0.03, p ​= ​0.98
Knee pain during activity (NRS, 0–10)	5.0 (2.2)	5.1 (2.2)	4.9 (2.4)	5.0 (2.1)	F(2,215) ​= ​0.27, p ​= ​0.76
KOOS Pain (0–100)	59.8 (17.4)	60.2 (15.5)	60.5 (17.7)	58.7 (18.7)	F(2,215) ​= ​0.24, p ​= ​0.78
KOOS Symptoms (0–100)	66.0 (17.2)	67.0 (15.4)	66.1 (17.3)	64.8 (18.8)	F(2,215) ​= ​0.30, p ​= ​0.74
KOOS ADL (0–100)	66.1 (18.5)	65.1 (17.7)	66.3 (17.8)	66.7 (20.2)	F(2,215) ​= ​0.13, p ​= ​0.88
KOOS Sport and recreation (0–100)	32.8 (23.5)	28.6 (21.1)	33.6 (24.0)	35.7 (24.9)	F(2,215) ​= ​1.64, p ​= ​0.20
KOOS QOL (0–100)	46.4 (17.4)	46.6 (18.3)	46.2 (16.4)	46.6 (17.8)	F(2,215) ​= ​0.01, p ​= ​0.99
ICOAP total (0–100)	30.0 (19.9)	30.9 (21.2)	31.1 (19.4)	27.9 (19.5)	F(2,215) ​= ​0.59, p ​= ​0.56
TSK, high kinesiophobia, n (%)^d^	74 (33.9)	20 (30.3)	29 (35.8)	25 (35.2)	X(2, N ​= ​218) ​= ​0.57, p ​= ​0.75
PASS, unacceptable, n (%)	101 (46.3)	35 (53.0)	35 (43.2)	31 (43.7)	X(2, N ​= ​218) ​= ​1.71, p ​= ​0.42
**Clinical features**					
Joint line tenderness, index median, n (%)	31 (14.2)	6 (9.1)	11 (13.6)	14 (19.7)	X(2, N ​= ​218) ​= ​3.21, p ​= ​0.2
Joint line tenderness, index lateral, n (%)	31 (14.2)	9 (13.6)	11 (13.6)	11 (15.5)	X(2, N ​= ​218) ​= ​0.14, p ​= ​0.93
Crepitus, index, n (%)	162 (74.3)	45 (68.2)	64 (79.0)	53 (74.6)	X(2, N ​= ​218) ​= ​2.24, p ​= ​0.33
**Laboratory features**					
IL-6, median [IQR]	3 [2,4]	3 [2,4]	3 [2,4]	2 [2,4]	X^2^(2, N ​= ​218) ​= ​4.37, p ​= ​0.11

Values are mean (SD) unless otherwise stated.

Abbreviations: ADL, activities of daily living; BMI, body mass index; cm, centimeter; ICOAP, intermittent and constant osteoarthritis pain; kg/m^2^, kilogram per square meter; KOOS, knee injury and osteoarthritis outcome score; MetS, Metabolic syndrome; Nm/kg, newton meter per kilogram; NRS, numerical rating scale; OA, osteoarthritis; PASS, patient acceptable symptom state; QOL, quality of life; SCQ, self-administered comorbidity questionnaire; TSK, Tampa Scale of Kinesiophobia.

Number of missing values ​> ​10: MetS. 17 participants have incomplete data for triglyceride measurements, making it impossible to calculate MetS for these individuals.

a The test statistics are shown for a continuous dependent variable using ANOVA: F(df1, df2), p-value or kruskal-wallis: X2(df1, N), p-value and for a categorical dependent variable using Chi-Square test: X(df1, N), p-value or Fisher's exact test: p-value.

b Presence of co-morbidities excluding knee osteoarthritis and overweight.

c Previous knee surgery for index and contralateral knee.

d A score >37 was considered to indicate a high level of kinesiophobia according to the TSK.

### 3.1 Structural features stratified by symptom duration

Table 2 shows the overall presence of structural features of KOA and the stratification by the duration of knee complaints. Of the 217 participants, 71% had TF OA and/or PF OA in their index knee. 114 participants (53%) had PF OA, 119 participants (55%) had TF medial OA, and 63 participants (29%) had TF lateral OA. No statistically significant differences were observed between the duration of knee complaints and structural features of knee OA, except for effusion-synovitis (X(2, N ​= ​217) ​= ​7.06, p ​= ​0.03). 4 participants had an anterior cruciate ligament reconstruction. An overview of the structural features of KOA stratified by planned or recent first consultation with the GP are presented in Table S7.

**Table 2:** Structural features of osteoarthritis stratified by duration of knee complaints.

	Overall (n ​= ​217^a^)	<12 months (n ​= ​65)	≥12-<24 months (n ​= ​81)	≥24 months (n ​= ​71)	Comparison
**OA**^b^	155 (71.4)	49 (75.4)	61 (75.3)	45 (63.4)	X(2, N ​= ​217) ​= ​3.35, p ​= ​0.19
**PF OA**	114 (52.5)	30 (46.2)	44 (54.3)	40 (56.3)	X(2, N ​= ​217) ​= ​1.58, p ​= ​0.45
Cartilage defect^c^	199 (91.7)	63 (96.9)	72 (88.9)	64 (90.1)	X(2, N ​= ​217) ​= ​3.4, p ​= ​0.18
Osteophyte	119 (54.8)	30 (46.2)	47 (58.0)	42 (59.2)	X(2, N ​= ​217) ​= ​2.85, p ​= ​0.24
BML	169 (77.9)	52 (80.0)	64 (79.0)	53 (74.6)	X(2, N ​= ​217) ​= ​0.66, p ​= ​0.72
**TF medial OA**	119 (54.8)	35 (53.8)	48 (59.3)	36 (50.7)	X(2, N ​= ​217) ​= ​1.16, p ​= ​0.56
Cartilage defect	160 (73.7)	50 (76.9)	60 (74.1)	50 (70.4)	X(2, N ​= ​217) ​= ​0.75, p ​= ​0.69
Osteophyte	138 (63.6)	41 (63.1)	55 (67.9)	42 (59.2)	X(2, N ​= ​217) ​= ​1.26, p ​= ​0.53
BML	114 (52.5)	37 (56.9)	42 (51.9)	35 (49.3)	X(2, N ​= ​217) ​= ​0.82, p ​= ​0.67
**TF lateral OA**	63 (29.0)	21 (32.3)	21 (25.9)	21 (29.6)	X(2, N ​= ​217) ​= ​0.73, p ​= ​0.69
Cartilage defect	147 (67.7)	48 (73.8)	51 (63.0)	48 (67.6)	X(2, N ​= ​217) ​= ​1.96, p ​= ​0.38
Osteophyte	111 (51.2)	29 (44.6)	45 (55.6)	37 (52.1)	X(2, N ​= ​217) ​= ​1.77, p ​= ​0.41
BML	72 (33.2)	25 (38.5)	21 (25.9)	26 (36.6)	X(2, N ​= ​217) ​= ​3.12, p ​= ​0.21
**Meniscus morphology**					
Medial	146 (67.3)	47 (72.3)	58 (71.6)	41 (57.7)	X(2, N ​= ​217) ​= ​4.37, p ​= ​0.11
Lateral	53 (24.4)	20 (30.8)	18 (22.2)	15 (21.1)	X(2, N ​= ​217) ​= ​2.05, p ​= ​0.36
**Effusion-synovitis**	72 (33.2)	30 (46.2)	22 (27.2)	20 (28.2)	**X(2, N = 217) = 7.06, p = 0.03**
**Hoffa-synovitis**	62 (28.6)	18 (27.7)	23 (28.4)	21 (29.6)	X(2, N ​= ​217) ​= ​0.06, p ​= ​0.97

Values show the number and percentage (n (%) of participants having the MRI OA feature. Abbreviations: BML, bone marrow lesion; OA, osteoarthritis; PF, patellofemoral; TF, tibiofemoral.

a One participant (<12-month group) did not have an MRI assessment, therefore, as a result, the final analysis included 217 participants. One participant had a patellectomy, therefore all features in the patella were not scored. PF OA was based on the presence of OA features in the trochlea of the femur.

b OA in the PF and medial and lateral TF compartment was defined using a previously proposed definition [28].

c Either partial or full thickness cartilage defect.

**Fig. 1:**
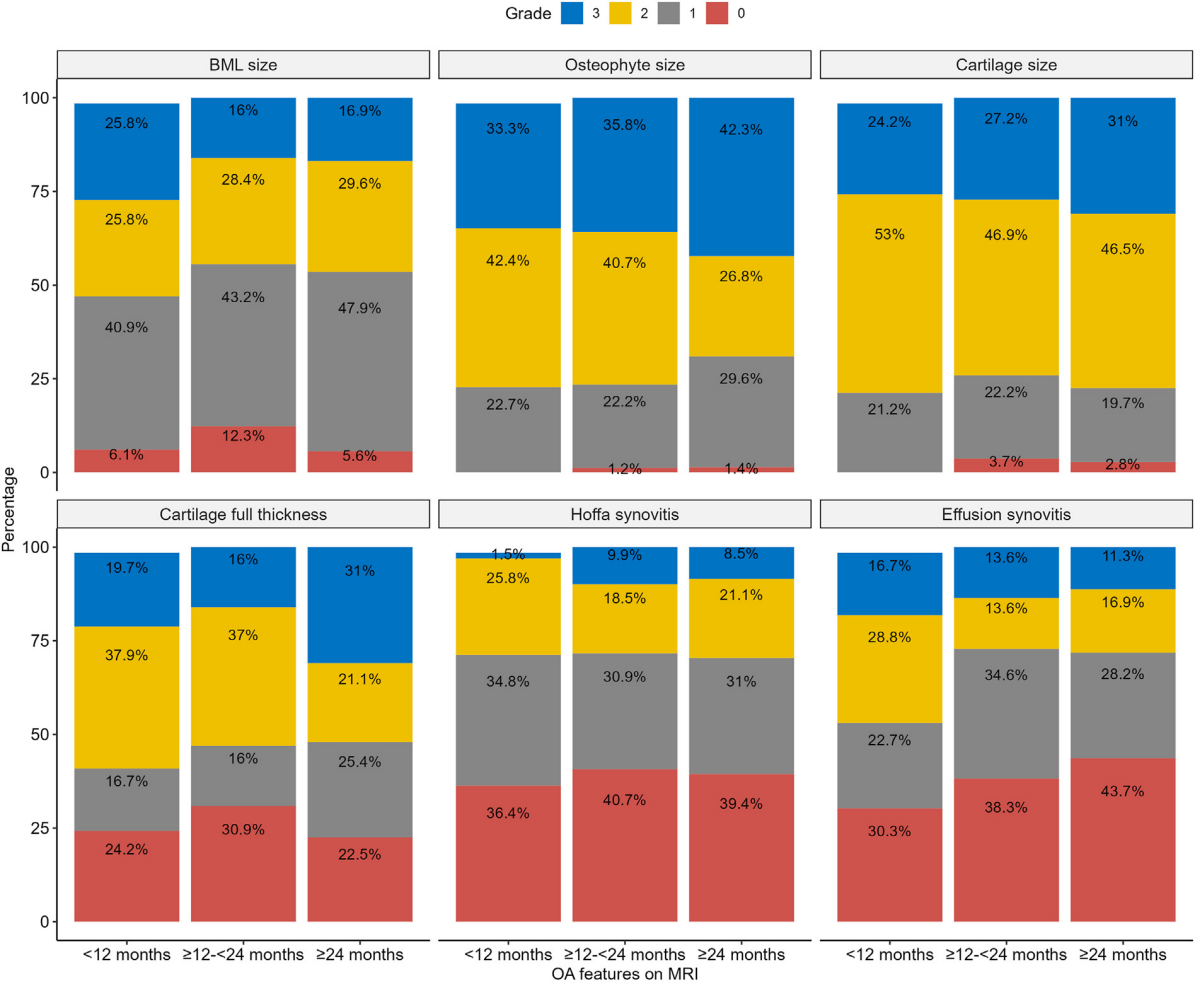
**Distribution of OA-related MRI features.** For BMLs, osteophytes, and cartilage, the highest grade of the subregions in the knee is presented.

Among the participants, 28% and 19% presented with grade 2 and 3 BMLs, respectively. Additionally, 37% and 37% presented with grade 2 and 3 osteophytes. 49% and 28% exhibited grade 2 and 3 cartilage damage, respectively, while 32% and 22% displayed grade 2 and 3 full-thickness cartilage damage. 22% and 7% presented with grade 2 and 3 Hoffa synovitis and 19% and 14% presented with grade 2 and 3 effusion synovitis. The distribution of these grading's stratified by the duration of knee complaints is illustrated in Fig. 1.

## 4 Discussion

This study aimed to enhance our understanding of the characteristics of a population with knee symptoms and overweight. This was done by describing the clinical and structural features of individuals presenting with knee complaints within 2 years of their initial primary care consultation or with an intention to visit. More than two thirds of individuals had MRI-defined structural KOA and almost half reported an unacceptable symptom state. Notably, the duration of knee complaints was not associated with the severity of symptoms nor the presence of structural KOA.

KOA is a progressive disease, whereby it is thought that the severity of symptoms typically increases with worsening of the disease as time passes [30]. However, our findings offer an alternative perspective, showing no association between the duration of knee complaints and symptom severity. This might partly be explained by the presence of more unpredictable intermittent pain patterns in early stages, making the relationship between duration and symptoms less prominent [31]. However, it's also important to consider that our study population likely reflects a population of individuals with more pronounced complaints, as individuals experiencing minimal symptoms might not have participated. Moreover, previous research has highlighted the inconsistencies between structural indicators and the severity of symptoms in OA, even in more advanced stages of the disease [[32], [33], [34]]. This discrepancy could be influenced by factors such as the definition of pain, the characteristics of the study population, and the definition of structural KOA. In fact, not all structural features of OA may be related to pain [[35], [36], [37]], emphasizing the importance of considering individual structural features of OA (like osteophytes, cartilage, and BMLs) rather than relying solely on a composite severity scores. Studies that focus on individual MRI features often report only their presence or absence, overlooking the severity or duration of symptoms and the extent of structural changes—factors crucial for understanding the state of the disease or illness [35]. Nevertheless, our findings do not reveal a clear distinction in specific structural features (i.e., osteophytes, cartilage, BML, meniscal morphology) when comparing different durations of knee complaints, with the exception of effusion-synovitis. The group with less than 12 months of knee complaints exhibited a higher prevalence of effusion-synovitis (including both synovitis and effusion). Though, accelerated KOA, characterized by rapid radiographic progression has been linked to synovitis [38]. Additionally, an increase in synovitis severity has been associated with cartilage deterioration over a two-year period [39]. However, given that synovitis can fluctuate over time [39], pinpointing an explanation using cross-sectional data is challenging. Further studies focusing on the earliest detectable MRI changes in KOA are required to more definitively understand the role of effusion synovitis in the early stages of the disease. Notably, a considerable number of individuals with less than a year of knee complaints showed significant cartilage damage, including many instances of full-thickness damage. Since multiple MRI features of KOA are found in the knees of participants who do not report pain [40], it could be that a concomitant presence of lesions may already exist once symptoms begin to emerge [41]. The duration of knee complaints is considered an important factor to distinguish early-stage KOA from its later stages, evident by its frequent use in study inclusion criteria [42]. However, our findings prompted important questions about the applicability and appropriate threshold values for symptoms duration for identifying individuals with early-stage KOA and overweight. This consideration is particularly important given the high severity of structural abnormalities, such as full-thickness cartilage damage, observed across all groups in our study.

All individuals had overweight, warranting consideration of its potential impact on KOA severity. While KOA is generally considered a slowly progressive disorder, it has been observed that a significant proportion of incident KOA cases, particularly among individuals with a history of knee injury, older people, or higher BMI, experience an accelerated onset and progression [43]. Specifically, in a community-based cohort, it was noted that 1 in 7 women with incident KOA, more likely among those with higher BMI, had accelerated KOA [44]. This tendency towards a faster progression in KOA, influenced by overweight, could explain the high prevalence of structural KOA in our study. Additionally, the presence of grade 2 and 3 OA features on MRI, even in individuals who have had knee complaints for less than two years, indicates a possibility for rapid progression in an early phase. An alternative explanation is that participants may have experienced an early and insidious onset of KOA, as other studies indicate that youth with obesity often exhibit pathological alterations associated with KOA [45,46]. However, given the cross-sectional nature of the data and the lack of historical body composition data, this hypothesis cannot be further examined. These findings suggest that primary care physicians should recognize that structural changes can already be present in individuals with overweight presenting with knee complaints, even if these complaints have been present for less than 12 months. Moreover, it is important to note that many participants in our study presented with at least two comorbidities, in addition to KOA and overweight. A greater number of comorbidities is linked with worse health indicators, including pain intensity, lower physical activity level, and quality of life [47]. Considering the likelihood of accelerated progression and more severe health outcomes, individuals with overweight may require distinct diagnostic criteria. Clinical features reflective of early-stage KOA in other groups may not be suitable to identify early stages in this population, who might already be presenting more advanced stages of the disease. Consequently, defining early KOA still requires careful consideration, as our findings within our studied population with overweight, may not be generalizable to other populations. However, given the prevalent link between KOA and overweight, our findings offer valuable insights for a significant proportion of individuals with KOA.

In this study, 71% of individuals exhibit MRI-defined KOA with an average knee pain severity at rest of 3.4 (2.4). In comparison, the CHECK cohort, which enrolled individuals within 0–6 months of their initial consultation for knee and/or hip complaints, 83% reported knee complaints with a mean hip and/or knee pain severity of 3.6 (2.1) over the past week, and a BMI of 26.2 ​± ​4.0 ​kg/m^2^ [48]. However, only 10% of this cohort had radiographic evidence of OA in one or both knees at baseline [49]. Comparing our results, the prevalence of MRI features in our study population exceeds those observed in the CHECK cohort, where 9% had TF OA on MRI at baseline, 60% exhibited some form of cartilage loss, 55% had at least one osteophyte, and 17% displayed a bone marrow lesions [50]. Similarly, among asymptomatic individuals over 40 years old, the pooled prevalence rates were 43% (29%–57%) for cartilage defects, 37% (22%–53%) for osteophytes, and 21% (14%–31%) for BMLs [51]. Despite similarities in demographics across studies, except for BMI, our findings emphasize a distinctly higher prevalence of structural KOA in our study population. Our study also indicates that the individuals who visited their GP experienced knee complaints for an average of 11 months before seeking healthcare, with 33% reporting knee complaints for more than two years at baseline. This finding seems comparable to the CHECK cohort, where, despite participants being within six months of their first consultation, 42% reported experiencing knee pain for longer than two years at baseline [48]. Among participants, 52% of those who had consulted their GP deemed their complaints unacceptable, compared to only 28% of those intending to consult, despite 62% of the latter having MRI-defined KOA. Additionally, the group intending to visit their GP reported lower knee pain severity (2.8 (2.4)), compared to those who had recently consulted (3.6 (2.4)).

When individuals, despite complaints, postpone consulting their GP, the opportunity for early treatment is diminished by time lost between symptom onset and diagnosis. It is possible that individuals do not perceive their symptoms as severe enough to warrant a visit to their GP, even though this group could benefit from treatment to prevent progression. Patients' healthcare-seeking behavior is complex and influenced by social, personal, cultural, economic, and experiential factors [52]. In the context of KOA, questions arise regarding how and when individuals decide that their knee complaints warrant consultation. Misconceptions about KOA as an untreatable aspect of aging or anticipation of negative responses from GPs may further delay consultation. In addition, coexisting health issues, particularly those with higher comorbidity burdens, might contribute to delayed consultation and/or early diagnosis as well. Evidence suggests that individuals rating their knee problems as a health priority are more likely to seek consultation [4]. Recognizing how comorbidities divert attention from knee complaints might be essential for early-stage KOA diagnosis in primary care, especially for individuals with overweight. Given these complexities, it is crucial to gather more detailed information at the time of an individual's initial presentation in primary care. This entails examining why individuals seek medical attention at that specific time, the symptoms they present with, and the GP's diagnostic reasoning in identifying early characteristics of KOA.

Several limitations of our study should be acknowledged. Although this is an explorative study, the reliance on multiple tests necessitates caution in interpreting the results. Relevant covariates exist but were not explicitly considered in the analysis. Moreover, the used OA definition presents challenges in assessing the severity of structural OA, as it only indicates the presence of absence of OA without gradations to reflect varying degrees of severity. To mitigate this, individual feature gradings were examined. Also, recalling the duration of complaints might be more challenging for individuals with longer symptom durations, potentially introducing recollection bias. Furthermore, the findings may not generalize to other countries, cultures, or healthcare systems where different factors may influence GP consultation. Our recruitment strategy likely captured a demographically diverse population with varying ages and socioeconomic statuses, potentially yielding more generalizable results. However, since recruitment was confined to the Rotterdam region, a geographical bias may have been introduced. Finally, there is a potential selection bias in our study population, as participants were individuals seeking help for their knee pain, and thus may not represent all individuals with early-stage KOA who have not consulted their GP.

## 5 Conclusion

This study confirms the diagnostic challenges in identifying stages of KOA among individuals with knee complaints and overweight, showing no clear association between the duration of knee complaints and symptoms severity or prevalence of structural KOA in a presumed early-stage population. Two thirds of all individuals exhibited MRI-defined structural KOA and half had an unacceptable symptom state within two years of their initial GP visit. This study contributes valuable insights into the role of knee complaint duration, that can help define diagnosis or classification criteria of early-stage KOA. Future research should focus on what factors influence timely consultation, as well as improving early detection and diagnostic strategies for early-stage KOA, specifically for individuals with overweight.

## Author contributions

All authors (NJ, DS, JR, EO, SB-Z, MvM) contributed to the study's conception and design as well as the interpretation of the data. NJ and MvM were involved in data collection, while NJ conducted the data analysis. NJ and EO participated in MRI data collection and analysis. The article was written by NJ. All other authors (DS, JR, EO, SB-Z, MvM) critically reviewed and edited the manuscript draft, ultimately approving the final manuscript.

## Funding

The work was supported by the 10.13039/501100001826Netherlands Organisation for Health Research and Development (ZonMW) (50-55515-98-004) and the Dutch 10.13039/501100000142Arthritis Society (10.13039/501100006315ReumaNederland) (ZNW 20–501). The funders had no role in study design, data collection, analysis, and interpretation of the data. The authors wish to acknowledge the Dutch 10.13039/501100000142Arthritis Society for the program grant provided for the Center of Excellence “OA Prevention and Early Treatment – OA Pearl”.

## Data availability statement

Data can be obtained upon reasonable request. Request should be directed towards Marienke van Middelkoop (m.vanmiddelkoop@erasmusmc.nl).

## Declaration of competing interest

The authors have declared no conflicts of interest.
