# Supplementary material for: Planned or recent first consultation with the general practitioner for knee complaints: Is it indicative of early-stage knee osteoarthritis?
Source: Osteoarthr Cartil Open. 2024 Jun 6;6(3):100493. doi: 10.1016/j.ocarto.2024.100493 (PMC11223112; doi:10.1016/j.ocarto.2024.100493)
Supplement: Multimedia component 1 [file mmc1.docx]

# Supplementary files

**File S1** MR Imaging protocol

MR imaging of the index knee was performed using a 1.5 Tesla MR unit (MAGNETOM Sola, Siemens Medical Solutions, Erlangen, Germany) and an 18-channel knee coil. The protocol consisted of the following sequences: (1) a sagittal, axial and coronal fast spin-echo proton density-weighted sequence with fat suppression, (2) a sagittal and axial proton density-weighted Dixon sequence with four types of images: fat-only, water-only, in-phase, and out-of-phase, and (3) a 3D fast spin-echo T2-weighted water excitation sequence.

| **Table S1** MRI sequence parameters | | | | | | |
| --- | --- | --- | --- | --- | --- | --- |
| **Sequence** | **Imaging plane** | **Slice thikness/spacing (mm)** | **Repetition time (ms)** | **Echo time (ms)** | **Field of view (mm)** | **Matrix** |
| PD FSE FS | Sagittal | 3.0/0.3 | 2600 | 42 | 160x160 | 400x400 |
| PD FSE FS | Coronal | 3.0/0.3 | 2080 | 43 | 160x160 | 400x400 |
| PD FSE FS | Axial | 3.0/0.3 | 2550 | 41 | 160x160 | 336x336 |
| PD Dixon | Sagittal | 3.0/0.3 | 2800 | 43 | 160x160 | 400x400 |
| PD Dixon | Axial | 3.0/0.3 | 2720 | 43 | 160x160 | 400x400 |
| 3D T2 FSE | Sagittal | 0.6/0.0 | 19.9 | 7.34 | 160x160 | 320x320 |
| *Abbreviations: FOV, field of view; FSE, fast spin-echo; PD, proton density; FS, fat suppression; SGE, spoiled gradient-echo; FIESTA, Fast Imaging Employing Steady-state Acquisition.* | | | | | | |

**File S2** gives an overview of the inter-observer reliability of the MOAKS scoring.

The researcher (NJ) who scored all MRIs of the knee was trained on MOAKS by an experienced musculoskeletal radiologist (EO., >15 years of experience). To assess inter-observer variability, fifteen cases were randomly selected and independently scored by two readers (NJ, EM). Kappa and Prevalence Adjusted Bias Adjusted Kappa (PABAK) statistics were utilized, with the calculations performed using dichotomized scores.

| **Table S2** Inter-observer reliability of MOAKS features | | | | | | | | | |
| --- | --- | --- | --- | --- | --- | --- | --- | --- | --- |
|  | **PF** | | | **TF Medial** | | | **TF Lateral** | | |
|  | **% agreement** | **KAPPA (95% CI)** | **PABAK (95% CI)** | **% agreement** | **KAPPA (95% CI)** | **PABAK (95% CI)** | **% agreement** | **KAPPA (95% CI)** | **PABAK (95% CI)** |
| Osteophytes | 0.77 | 0.18 (-0.13, -.49) | 0.53 (0.36, 0.71) | 0.80 | 0.54 (0.27, 0.81) | 0.60 (0.37, 0.83) | 0.89 | 0.75 (0.54, 0.96) | 0.78 (0.59, 0.96) |
| Cartilage | 0.75 | 0.40 (0.13, 0.66) | 0.50 (0.28, 0.72) | 0.69 | 0.36 (0.14, 0.57) | 0.39 (0.18, 0.60) | 0.72 | 0.40 (0.18, 0.62) | 0.44 (0.24, 0.64) |
| BML | 0.93 | 0.85 (0.71, 0.99) | 0.87 (0.74, 0.99) | 0.81 | 0.52 (0.30, 0.75) | 0.63 (0.45, 0.80) | 0.91 | 0.54 (0.22, 0.86) | 0.81 (0.68, 0.95) |
| *Abbreviations: BML, bone marrow lesions; PABAK, Prevalence adjusted bias adjusted kappa; PF, patellofemoral; TF, tibiofemoral.* | | | | | | | | | |

**File S3** Definition of structural knee osteoarthritis

Tibiofemoral (TF) OA was defined using a previously proposed definition as the presence of a definite osteophyte and full-thickness cartilage loss, or one of these features and two of the following features: (1) subchondral bone marrow lesion or cyst not associated with meniscal or ligamentous attachments, (2) meniscal subluxation, maceration or degenerative tearing (such as a horizontal tear), or (3) partial-thickness cartilage loss. Patellofemoral OA was defined as the presence of a definite osteophyte and either partial or full thickness cartilage loss in the patella or anterior femur. Although MOAKS does not encompass scoring of bone attrition the presence of two out of the three remaining features was still considered necessary to define KOA.

**Table S4** gives an overview of the presence, the treatment of, and activity limitations of co-morbidities in participant with KOA and overweight or obesity.

| **Table S4** Co-morbidities in participants with knee osteoarthritis and overweight or obesity | | | |
| --- | --- | --- | --- |
| **Condition** | **Presence** | **Treatment** | **Limits activities** |
| Heart Disease | 15 (6.9) | 11 (73.3) | 1 (6.7) |
| High Blood Pressure | 64 (29.4) | 46 (71.9) | 4 (6.2) |
| Lung Disease | 29 (13.3) | 18 (62.1) | 12 (41.4) |
| Diabetes | 15 (6.9) | 14 (93.3) | 2 (13.3) |
| Ulcer or stomach disease | 8 (3.7) | 5 (62.5) | 1 (12.5) |
| Kidney disease | 4 (1.8) | 1 (25.0) | 1 (25.0) |
| Liver disease | 3 (1.4) | 3 (100.0) | 3 (100.0) |
| Anemia or other blood disease | 5 (2.3) | 4 (80.0) | 1 (20.0) |
| Cancer | 6 (2.8) | 5 (83.3) | 4 (66.7) |
| Depression | 8 (3.7) | 5 (62.5) | 4 (50.0) |
| Elbow complaints | 17 (7.8) | 1 (5.9) | 9 (52.9) |
| Hand complaints | 83 (38.1) | 9 (10.8) | 33 (39.8) |
| Rheumatoid arthritis | 11 (5.0) | 1 (9.1) | 6 (54.5) |
| Neck complaints | 59 (27.1) | 17 (28.8) | 20 (33.9) |
| Headache or migraine | 44 (20.2) | 6 (13.6) | 15 (34.1) |
| Neurological condition | 8 (3.7) | 5 (62.5) | 4 (50.0) |
| Other condition | 65 (29.8) | 31 (49.2) | 21 (33.3) |
| Second other condition | 11 (5.0) | 1 (9.1) | 4 (36.4) |
| *Values show the number and percentage (n (%)) of participants having the co-morbidity, receiving treatment for it, or limiting their activities.* | | | |

**Table S5** provides an overview of the self-reported prevalence of OA in joints other than the knee, including the neck, hip, back, hands and/or fingers, ankles, shoulders, and feet and/or toes.

| **Table S5** Self-reported prevalence of OA in joints other than the knee | |
| --- | --- |
| **Joint** | **Prevalence (N=218)** |
| Any joint | 80 (36.7) |
| Neck | 17 (7.8) |
| Hip | 21 (9.6) |
| Back | 16 (7.3) |
| Hand and/or fingers | 52 (23.9) |
| Ankle | 13 (6.0) |
| Shoulder | 18 (8.3) |
| Feet and/or toes | 21 (9.6) |
| *Values show the number and percentage (n (%)) of participants having OA in that joint. Participants responded to a multiple-choice question, "In which other joints besides your knee do you have arthritis?"* | |

**Table S6** provides and overview of the characteristics of the study population stratified by participants who either intended to consult their general practitioner with knee complaints for the first time regarding their current complaints or already did so within the previous 24 months

| **Table S6** Characteristics of the study population stratified by planned or recent first consultation with the general practitioner | | |
| --- | --- | --- |
| **Characteristics** | **Planned consultation (n=53)** | **Recent consultation (n=165)** |
| **Demographics** | | |
| Sex, n (%) |  |  |
| Female | 32 (60.4) | 110 (66.7) |
| Male | 21 (39.6) | 54 (32.7) |
| Intersex | 0 (0.0) | 1 (0.6) |
| Age, years | 58.91 (6.81) | 59.10 (6.01) |
| BMI, kg/m^2^ | 32.97 (4.83) | 31.48 (4.39) |
| Co-morbidities* | 2.15 (1.66) | 2.07 (1.82) |
| MetS, n (%) | 16 (30.2) | 46 (31.1) |
| Education, n (%) |  |  |
| Primary or secondary | 9 (17.0) | 42 (25.5) |
| Vocational | 19 (35.8) | 58 (35.2) |
| Higher | 22 (41.5) | 63 (38.2) |
| Other | 3 (5.7) | 2 (1.2) |
|  | | |
| Unilateral knee complaints, n (%) | 23 (43.4) | 108 (65.5) |
| Previous knee surgery**, n (%) | 4 (7.5) | 27 (16.4) |
|  | | |
| Knee pain at rest (NRS, 0-10) | 2.75 (2.43) | 3.58 (2.35) |
| Knee pain during activity (NRS, 0-10) | 4.08 (1.99) | 5.25 (2.21) |
| KOOS Pain (0-100) | 66.72 (13.53) | 57.59 (17.90) |
| KOOS Symptoms (0-100) | 72.30 (14.06) | 63.94 (17.64) |
| KOOS ADL (0-100) | 72.00 (16.51) | 64.17 (18.77) |
| KOOS Sport and recreation (0-100) | 41.42 (23.74) | 30.03 (22.84) |
| KOOS QOL (0-100) | 52.59 (14.42) | 44.47 (17.80) |
| ICOAP total (0-100) | 22.68 (16.40) | 32.33 (20.41) |
| TSK, high kinesiophobia, n (%)*** | 15 (28.3) | 59 (35.8) |
| PASS, unacceptable, n (%) | 15 (28.3) | 86 (52.1) |
|  | | |
| Joint line tenderness, index median, n (%) | 6 (11.3) | 25 (15.2) |
| Joint line tenderness, index lateral, n (%) | 8 (15.1) | 23 (13.9) |
| Crepitus, index, n (%) | 36 (67.9) | 126 (76.4) |
|  | | |
| IL-6, median [IQR] | 3 [2, 3] | 3 [2, 4] |
| *Values are mean (SD) unless otherwise stated. Abbreviations: ADL, activities of daily living; BMI, body mass index; cm, centimeter; ICOAP, intermittent and constant osteoarthritis pain; kg/m^2^, kilogram per square meter; KOOS, knee injury and osteoarthritis outcome score; MetS, Metabolic syndrome; Nm/kg, newton meter per kilogram; NRS, numerical rating scale; OA, osteoarthritis; PASS, patient acceptable symptom state; QOL, quality of life; SCQ, self-administered comorbidity questionnaire; TSK, Tampa Scale of Kinesiophobia.*  **Presence of co-morbidities excluding knee osteoarthritis and overweight.*  ***Previous knee surgery for index and contralateral knee.*  ****A score >37 was considered to indicate a high level of kinesiophobia according to the TSK.*  *Number of missing values >10: MetS. 17 participants have incomplete data for triglyceride measurements, making it impossible to calculate MetS for these individuals.* | | |

**Table S7** provides and overview of the structural features of osteoarthritis stratified by participants who either intended to consult their general practitioner with knee complaints for the first time regarding their current complaints or already did so within the previous 24 months.

| **Table S7** Structural features of osteoarthritis stratified by planned or recent first consultation with the general practitioner | | |
| --- | --- | --- |
|  | **Planned consultation (n=53)** | **Recent consultation (n=165)** |
| **OA**** | 32 (61.5) | 123 (74.5) |
| **PF OA** | 21 (40.4) | 93 (56.4) |
| Cartilage defect*** | 49 (94.2) | 150 (90.9) |
| Osteophyte | 21 (40.4) | 98 (59.4) |
| BML | 41 (78.8) | 128 (77.6) |
| **TF medial OA** | 25 (48.1) | 94 (57.0) |
| Cartilage defect | 31 (59.6) | 129 (78.2) |
| Osteophyte | 28 (53.8) | 110 (66.7) |
| BML | 23 (44.2) | 91 (55.2) |
| **TF lateral OA** | 13 (25.0) | 50 (30.3) |
| Cartilage defect | 34 (65.4) | 113 (68.5) |
| Osteophyte | 19 (36.5) | 92 (55.8) |
| BML | 17 (32.7) | 55 (33.3) |
| **Meniscus morphology** | | |
| Medial | 33 (63.5) | 113 (68.5) |
| Lateral | 9 (17.3) | 44 (26.7) |
| **Effusion-synovitis** | 9 (17.3) | 63 (38.2) |
| **Hoffa-synovitis** | 10 (19.2) | 52 (31.5) |
| *Values show the number and percentage (n (%) of participants having the MRI OA feature. Abbreviations: BML, bone marrow lesion; OA, osteoarthritis; PF, patellofemoral; TF, tibiofemoral.*  **One participant (< 12-month group) did not have an MRI assessment, therefore, as a result, the final analysis included 217 participants. One participant had a patellectomy, therefore all features in the patella were not scored. PF OA was based on the presence of OA features in the trochlea of the femur.*  ***OA in the PF and medial and lateral TF compartment was defined using a previously proposed definition.[28]*  ****Either partial or full thickness cartilage defect.* | | |
